# Supplementary material for: The interactome of intact mitochondria by cross-linking mass spectrometry provides evidence for coexisting respiratory supercomplexes
Source: Mol Cell Proteomics. 2017 Dec 8;17(2):216–32. doi: 10.1074/mcp.RA117.000470 (PMC5795388; doi:10.1074/mcp.RA117.000470)
Supplement: Supplemental Data [file supp_RA117.000470_133922_0_supp_23978_fzffwf.zip › spectra_annotation/mito_DR_spectra_annotation/445-1-3-1-16-1.pdf]

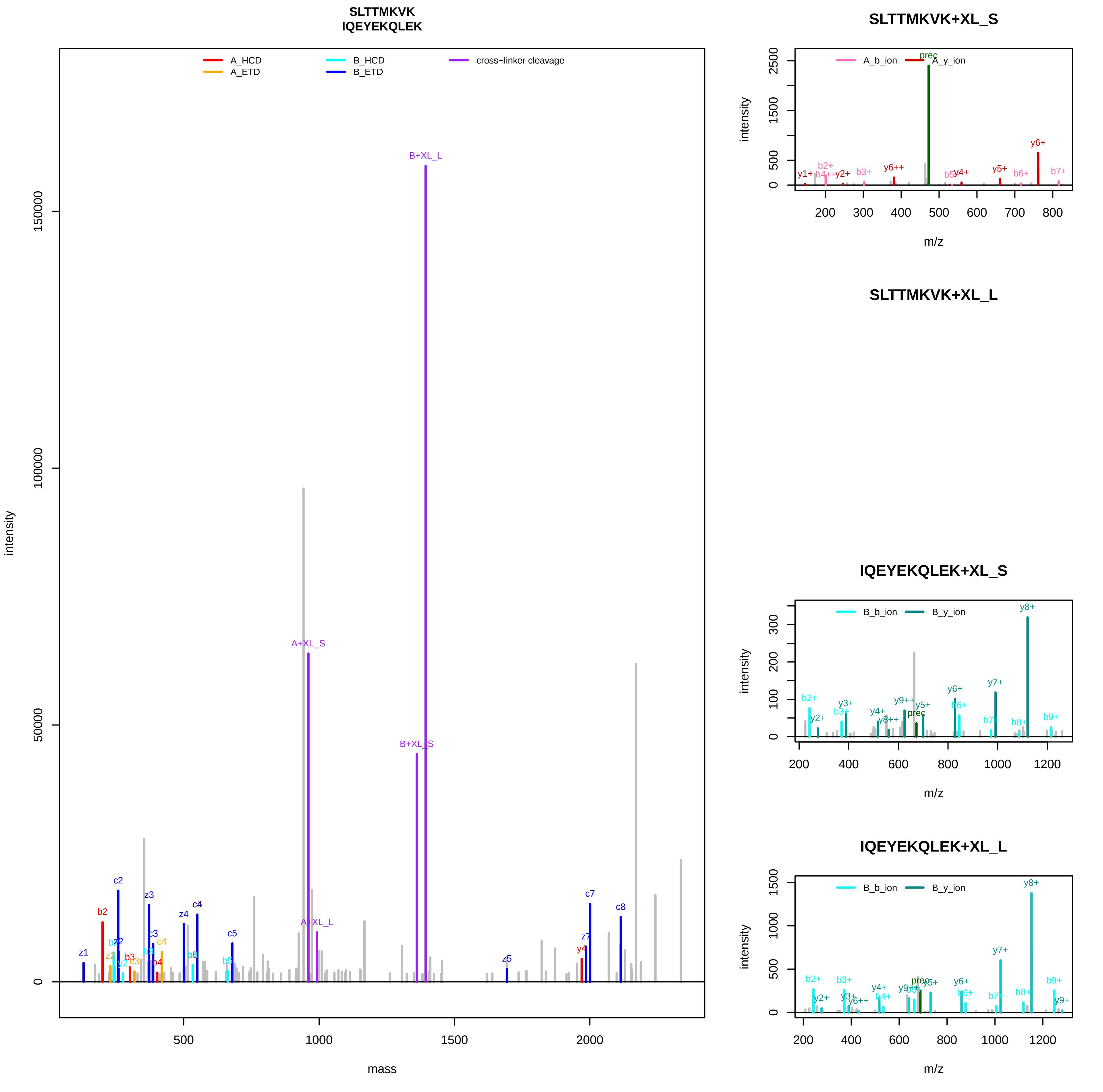
SLTTMKVK
IQEYEKQLEK
A_HCD
A_ETD
B_HCD
B_ETD
cross−linker cleavage
B+XL_L
A+XL_S
B+XL_S
A+XL_L
intensity
mass
0
50000
100000
150000
500
1000
1500
2000
z1
b2
c2
z3
z4
c4
c5
z5
z7
c7
c8
y4
SLTTMKVK+XL_S
A_b_ion
A_y_ion
prec
y6+
y6++
y5+
y4+
y2+
y1+
b2+
b3+
b5+
b6+
b7+
m/z
SLTTMKVK+XL_L
IQEYEKQLEK+XL_S
B_b_ion
B_y_ion
y8+
y7+
y6+
y5+
y4+
y3+
y2+
y9++
y8++
b2+
b3+
b6+
b7+
b8+
b9+
IQEYEKQLEK+XL_L
